# Effect of pedestal fan use on serum stress biomarkers in older adults exposed to simulated daylong indoor overheating

**DOI:** 10.14814/phy2.70390

**Published:** 2025-06-21

**Authors:** Ben J. Lee, Thomas McCarthy, Fergus O'Connor, Sarah L. Davey, C. Douglas Thake, James J. McCormick, Kelli E. King, Pierre Boulay, Robert D. Meade, Glen P. Kenny

**Affiliations:** ^1^ Occupational and Environmental Physiology Group, Centre for Physical Activity, Sport, Exercise Sciences Coventry University Coventry UK; ^2^ Human and Environmental Physiology Research Unit, School of Human Kinetics University of Ottawa Ottawa Ontario Canada; ^3^ Faculty of Physical Activity Sciences University of Sherbrooke Sherbrooke Quebec Canada; ^4^ Department of Epidemiology, T.H. Chan School of Public Health Harvard University Boston Massachusetts USA; ^5^ Clinical Epidemiology Program Ottawa Hospital Research Institute Ottawa Ontario Canada

**Keywords:** aging, climate change, electric fans, enterocyte damage, immune response, inflammation

## Abstract

There is an urgent need to develop targeted heat‐alleviation strategies to protect heat‐vulnerable older adults. We have shown that electric fan use had nominal impact on reducing body core temperature and cardiovascular strain during daylong exposure to simulated indoor overheating. Here, we examined the effects of pedestal fan use during exposure to hot conditions on systemic markers of enterocyte damage, immune activation, renal ischemia, and inflammation in older adults. Eighteen adults (8 females; age 72, SD 7 years) underwent 3 randomized 8‐h exposures to 36°C, 45% relative humidity seated in front of a fan delivering air speeds of 0 m/s (control), 2 m/s (normal air speed delivered by electric fans), or 4 m/s (air speed used in biophysical modeling). Body core temperature and cardiovascular strain were measured throughout. Blood samples were obtained for analysis of systemic biomarkers before and at the end of exposure. End‐exposure core temperature was elevated ~1.0°C from baseline in all conditions. Following heat exposure, IFABP increased by 364 pg/mL [95% CI: 59, 670; *p* = 0.02] and LBP increased by 3.06 ng/mL [1.12, 5.00; *p* = 0.002] but was not different between the fan use and control condition (all *p* ≥ 0.15). No changes were observed for sCD14, IL‐6, TNFα, CRP, or NGAL (all *p* ≥ 0.15). The use of electric fans during daylong exposure to indoor overheating failed to meaningfully mitigate increases in physiological strain or biochemical markers associated with enterocyte damage and immune activation in older adults. ClinicalTrials.gov identifier: NCT05695079.

## Graphical abstract

Pedestal fans were not effective at mitigating physiological strain or indices of gastrointestinal barrier damage and endotoxemia in older adults exposed to daylong indoor overheating.

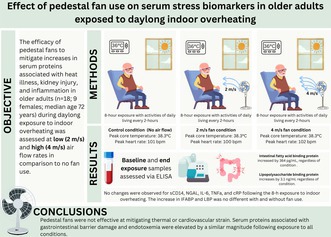

## 1 INTRODUCTION

The escalating frequency and intensity of heatwaves pose significant health risks, particularly to heat vulnerable sectors of the population (Ebi et al., 2021). Older adults are among the most at risk, especially those with age‐associated chronic conditions linked with heat vulnerability (e.g., cardiovascular disease, type 2 diabetes, obesity) (Kenny et al., 2010, 2024; Semenza et al., 1999; Vandentorren et al., 2004). The increased risk in older adults has been attributed to age‐associated impairments in body temperature regulation secondary to blunted increases in skin blood flow and sweating (Kenny et al., 2010; Meade et al., 2020). The impaired regulation of body temperature alongside declines in central haemodynamic responses supporting thermoregulation during heat stress (e.g., elevations in cardiac output) is thought to contribute to dangerous elevations in body core temperature, circulatory strain, and systemic inflammation during heat exposure, increasing the risk for numerous adverse health outcomes (e.g., heat stroke, major adverse cardiovascular events, acute kidney injury) (Meade et al., 2020; Meade, Notley, Akerman, McGarr, et al., 2023). Developing evidence‐based guidance on effective interventions for alleviating the physiological burden of extreme heat in vulnerable populations, such as older adults, is therefore a public health priority and key component of heat‐health adaptation planning (Jay et al., 2021).

While air‐conditioning provides the most effective protection from extreme heat, it is inaccessible for many individuals (Hansen et al., 2011). Air‐conditioning is also energy intensive, can strain the electrical grid and, depending on the source of electricity generation, contribute to increasing greenhouse gas emissions, and contribute to the urban heat island effect (Dong et al., 2021; Patz et al., 2005). For these reasons, recent guidance has advocated the use of electric fans as a simple and sustainable alternative to air‐conditioning. Based on data in young adults and biophysical modeling, electric fans have been proposed as an effective and accessible cooling intervention for older adults (Morris et al., 2019, 2021). However, a recent reanalysis of these data indicated that fans provide minimal body cooling above 33°C in both young and older adults (Meade, Notley, et al., 2024). In support of this reanalysis, we have demonstrated that while electric fan use increases perceptions of well‐being (O'Connor et al., 2025) and does not exacerbate heat strain in hot environments, they do not meaningfully mitigate increases in body core temperature or cardiovascular strain, regardless of air velocity (2 m/s and 4 m/s) (O'Connor et al., 2024).

One limitation of most previous research on fan use is that analysis of body core temperature and cardiovascular strain alone does not capture the full physiological consequences of extreme heat exposures (Chin & Mackinnon, 2006). For example, the dual pathway model of heat stroke postulates that heat intolerance results from a transient shift in physiological state in the form of immune disturbances that promote endotoxemia and systemic inflammation, suppressing anti‐lipopolysaccharide (LPS) mechanisms, and potentially leading to heat illness and injury (Chin & Mackinnon, 2006). The initial step in this cascade of cellular events involves the breakdown of the gastrointestinal barrier, and subsequent leakage of bacterial endotoxins into the systemic circulation (Dokladny et al., 2015). We and others have previously shown that older adults experience greater intestinal enterocyte damage, as evidenced by increases in intestinal fatty acid binding protein (IFABP) concentrations (Foster et al., 2023; Lee, Russell, et al., 2024). Furthermore, we observed this response to be greater in temperatures over 31°C, relative to safe indoor temperatures (i.e., ≤26°C) recommended in public health guidance (Lee et al., 2025a). Despite the observed increase in IFABP, we did not observe increases in soluble cluster of differentiation 14 (sCD14), an acute phase protein associated with monocyte activation (Maliszewski & Wright, 1979; Wright et al., 1990), or downstream inflammatory proteins (Lee et al., 2025a).

To date, only one study has examined the efficacy of electric fan use to modulate systemic stress responses (Lei et al., 2025). This experiment demonstrated that electric fans failed to mitigate the systemic inflammatory response, secondary to damage to intestinal enterocytes in healthy young adults (~25 years old) exposed to uncompensable heat stress conditions (40°C, 55% RH) for 8 h, despite reductions in body core temperature (Lei et al., 2025). However, to the best of our knowledge, no study has assessed the effect of fans on intestinal enterocyte damage, immune activation, acute kidney injury, and systemic inflammation in older adults exposed to conditions more commonly experienced during hot weather and heat waves. Considering the increased heat vulnerability in older adults, this remains an important area of study. We evaluated the effectiveness of different fan speeds in modulating intestinal enterocyte damage, immune activation, systemic inflammation, and a surrogate measurement of renal ischemia in older adults (>65 years old) during a prolonged exposure to simulated overheating. The present study is exploratory in nature; therefore, no formal hypothesis is stated. However, considering our primary report indicated that fans provided limited mitigations to both body core temperature increases and cardiovascular strain, we felt it unlikely that any beneficial effects would be demonstrated on systemic stress biomarkers.

## 2 METHODS

### 2.1 Participants

**FIGURE 1:**
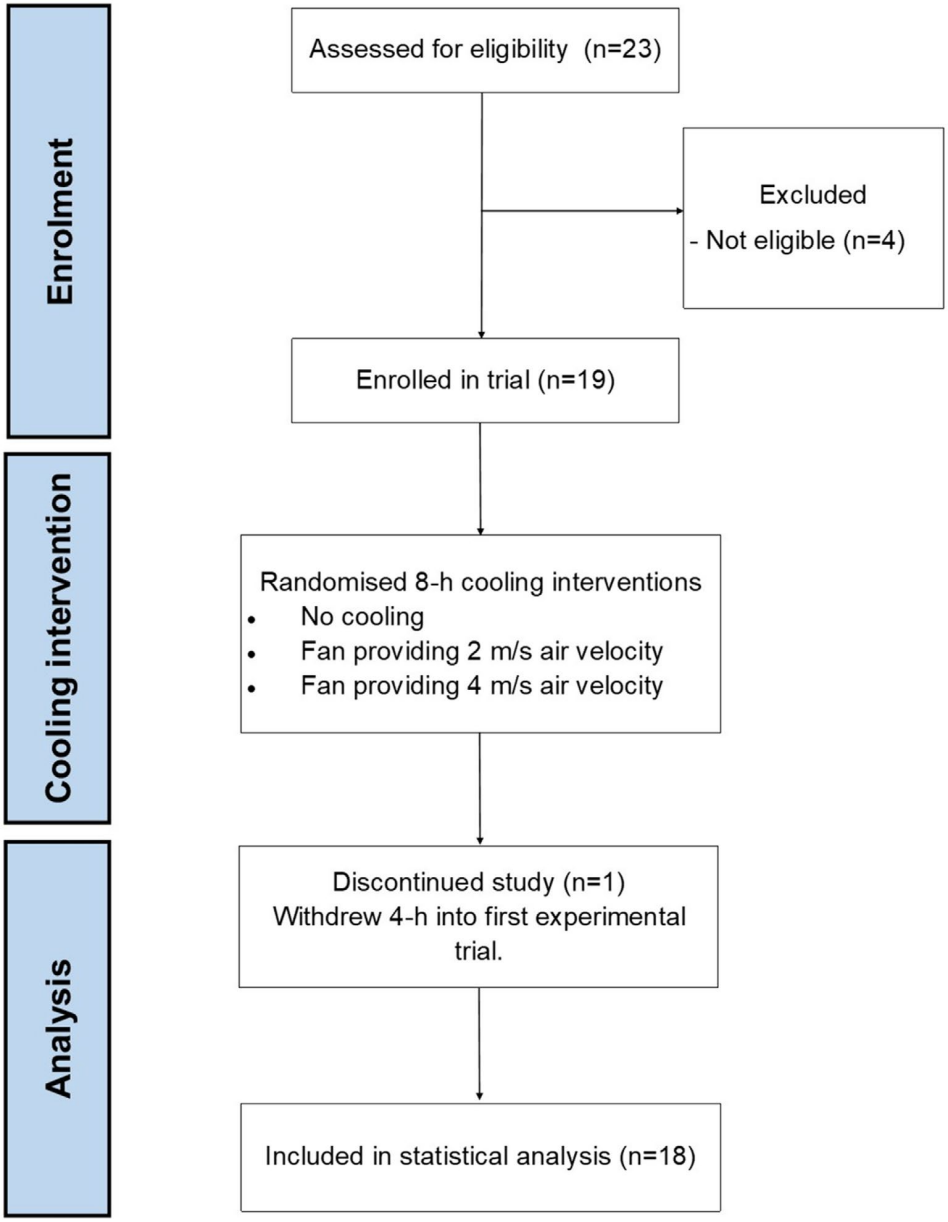
Flow of participants through the study. A CONSORT diagram depicting participant enrollment, randomization, and analysis. Out of 23 individuals screened for eligibility, 18 adults were enrolled. All completed 3, randomized 8 h heat exposures with: No cooling (control), fan at 2 m/s, or fan at 4 m/s.

The present study was part of a larger investigation evaluating electric fan use in older adults; the procedures of which are described in detail elsewhere (O'Connor et al., 2024). Following approval from the University of Ottawa Research Ethics Committee (H‐11‐21‐7572), 19 adults aged 65–85 years old from Ontario, Ottawa, Canada volunteered and provided their written informed consent prior to completing in the study. This study followed the EQUATOR guidelines for the reporting of randomized controlled trials (CONSORT) and was conducted in accordance with the Declaration of Helsinki. Prospective participants were eligible if they were 65–85 years old, nonsmoking, spoke English or French, and were able to provide informed consent (both males and females were eligible). Exclusion criteria included physical restriction (e.g., due to disease: intermittent claudication, renal impairment, active proliferative retinopathy, cardiovascular or pulmonary disease, severe arthritis, etc.), use of or changes in medication judged by the volunteers physician or investigators to make participation in this study inadvisable (e.g., medications suggested to increase risk of heat‐related illness; beta blockers, anticholinergics), cardiac abnormalities identified via 12‐lead ECG during an incremental exercise test to volitional fatigue (performed for all participants), a peak aerobic capacity (V̇O_2peak_), as measured during an incremental exercise test to volitional fatigue, exceeding the 50th percentile of age‐ and sex‐specific normative values published by the American College of Sports Medicine (ACSM). Participant characteristics of those enrolled are provided in Table 1, and participant flow through the study presented in Figure 1.

**TABLE 1:** Physical characteristics of participants that completed the experiment.

Variable	All participants (*n* = 18)^a^
Age, y	72 (67–76)
Sex	
Female	8 (44%)
Male	10 (56%)
Height, cm	170 (160–176)
Mass, kg	75.0 (60.5–83.5)
Body mass index^b^	25.7 (23.4–28.4)
Body surface area, m^2^ ^c^	1.9 (1.7–2.0)
Smoking status^d^	
Never	12 (67%)
Past	6 (33%)
Habitual physical activity, min/wk^e^	164 (75–191)
Types of physical activity^e^	
Walking	12 (67%)
Jogging, biking, swimming, rowing	3 (17%)
Organized sports	2 (11%)
Peak oxygen consumption, mL/kg/min^f^	24 (20–28)
ACSM V̇O_2peak_ percentile, %^f^	18 (9–31)
Using prescribed medications^g^	16 (89%)
Heat vulnerability linked medications^g^	
Antihypertensives	8 (44%)
Antidepressants	4 (22%)
Anthihyperglycemic	2 (11%)
Hemoglobin A_1C_ ^h^, %	5.7 (5.3–6.0)

Abbreviations: ACSM, American College of Sports Medicine; V̇O_2peak_, peak oxygen consumption.

^a^ Values are median and interquartile range or number of participants (percentage).

^b^ Calculated as weight in kilograms divided by height in meters squared.

^c^ Body surface area estimated using the Du Bois equation: 0.2047 × height in meters^0.725^ × mass in kg^0.425^.

^d^ Smoking status determined via participant self‐report. Prospective participants were excluded if they presently smoked. All those who previously smoked quit >19 years prior to participation.

^e^ Participant self‐reported physical activity level determined using the Canadian Society for Sport and Exercise Get Active Questionnaire. The types of physical activity performed were determined using the Kohl Physical Activity Questionnaire.

^f^ Prospective participants V̇O_2peak_ was assessed during an incremental cycling test to volitional fatigue. Participants were excluded if their V̇O_2peak_ exceeded the 50th percentile of age‐and‐sex specific normative data published by ACSM.

^g^ Participant prescription medication use was determined by self‐report. Some participants reported taking medications that have been *suggested* to increase heat vulnerability (e.g., antidepressants) or are associated with health conditions known to reduce heat tolerance (e.g., type 2 diabetes, heart disease). Reported medications included antihypertensives (*n* = 8), antidepressants (*n* = 4), metformin (*n* = 2), statins (*n* = 8), topical creams/ointments (*n* = 4), corticosteroids (*n* = 2), bronchodilators (*n* = 2), antihistamines (*n* = 3), and medications treating hyperactive thyroid (*n* = 2), occasional pain (*n* = 2) and osteoporosis (*n* = 3).

^h^ Hemoglobin A1c, Glycated hemoglobin.

### 2.2 Preliminary screening

Before attending the screening visit, participants completed the Canadian Society for Exercise Physiology (CSEP) Get Active Questionnaire (GAQ) and the American Heart Association Pre‐Participation Screening Questionnaire to assess their eligibility to participate. Habitual physical activity levels were also evaluated using the GAQ and the Kohl Physical Activity Questionnaire (Kohl et al., 1988). Participants who provided informed consent and met the inclusion criteria were familiarized with all aspects of the trial and measurements.

Anthropometric data, including height (model 2391, Detecto, Webb City, MO, USA) and body mass (IND560, Mettler Toledo Inc., Mississauga, ON, Canada), were collected and used to calculate body mass index and body surface area (Du Bois, 1989). Participants then underwent an incremental exercise protocol (CSEP, 1986) to the point of volitional fatigue on a semi‐recumbent cycle ergometer (Corival, Lode B.V., Groningen, the Netherlands) in thermoneutral conditions (∼22°C). Electrocardiogram was monitored by a certified exercise physiologist, using a 12‐lead echocardiogram. Peak aerobic capacity was measured via indirect calorimetry using an automated metabolic gas analysis system (MCD Medgraphics Ultima Series, MGC Diagnostics, MN, USA).

### 2.3 Experimental overview

On separate days, participants completed 3 randomized 8‐h exposures to 36°C, 45% relative humidity – conditions reflective of recent extreme heat events experienced in North America and which have been shown to elicit damage to the intestinal enterocyte layer in older adults (Lee et al., 2025a). Conditions were separated by a minimum of 6 days. Randomization was determined via a simple randomization key produced with Microsoft® Excel®. Participants were seated while facing forward in front of a fan, situated 1.0 m away, delivering air speeds of 0 m/s (control), 2 m/s (reflecting typical air speeds delivered by commercial electric fans; Morris et al., 2019), and 4 m/s (reflecting air speeds used in recent biophysical modeling; Meade, Notley, et al., 2024). Participants and researchers were blinded to the fan conditions until arrival at the laboratory. Biochemistry data were unblinded after statistical analysis. At the end of hours 1, 3, 5, and 7, participants performed 10 min of light stepping (~2.25 metabolic equivalents) to simulate activities of daily living. Drinking water (~20°C) was available ad libitum throughout.

### 2.4 Preexposure instructions and standardization

To minimize potential seasonal acclimatization effects, all experiments were conducted in Winter (December 2022) through to early spring (March 2023). Before each visit, participants were instructed to avoid intense physical activity, alcohol consumption (24 h prior), and excessive caffeine intake (12 h prior, no more than their habitual intake). They were also advised to drink ∼500 mL of water the night before and morning of each visit. Participants arrived between 07:00 and 09:00, with arrival times consistent within participants to minimize the effects of circadian variation. Participants were instructed to eat a light meal 2 h before each laboratory visit. During the baseline measurement period for each indoor exposure simulation, participants were asked a series of questions regarding heat exposure, physical activity, and sleep habits, meals, and general well‐being in the days leading up to the session. This information was recorded using a custom form and emailed to the participant ∼48 h prior to the subsequent experimental exposures. The participant was asked to follow the activity pattern for the previous experimental exposure as closely as possible before each laboratory visit.

### 2.5 Simulated heatwave exposure

On arrival to the laboratory, hydration status was confirmed via measurement of urine specific gravity (≤1.025; Reichert TTS 400, Reichert, Depew, USA) (Kenefick & Cheuvront, 2012), and the participant inserted a rectal thermocouple temperature probe ~12 cm past the anal sphincter. Following a measurement of nude body mass to an accuracy of 10 g using a high‐performance digital weighing terminal (model CBU150X; Mettler Toledo Inc.), participants dressed in light summer clothing (sandals, shorts, and a light shirt). Thereafter, the participant rested in a seated posture (reclined slightly) in a ∼22°C room adjacent to the climate chamber for ∼30 min. During this time, participants were instrumented with digital skin temperature sensors (DS1922L Thermochron, OnSolution Pty Ltd., Sydney, Australia) affixed to eight body regions, using double‐sided adhesives and medical tape. Mean skin temperature was calculated according to the weightings recommended in ISO 9886:2004: 7% forehead, 17.5% right scapula, 17.5% upper left chest, 7% upper right arm, 7% right forearm, 5% left hand, 19% right anterior thigh, and 20% left calf. During the instrumentation period, participants answered a series of questions on activity patterns in the days leading up to the trial (described Section 2.4). Baseline measurements were then recorded and a venous blood sample obtained from an antecubital vein (described in Section 2.6).

Participants were then transferred to a chamber regulated to 36°C, 45% relative humidity (heat index: 41°C) to simulate peak indoor temperatures during heat events when ambient cooling (e.g., air conditioning) is unavailable or otherwise not used. This condition is consistent with indoor temperatures measured in non‐air‐conditioned houses and during the 2021 heat dome event in the North American Pacific Northwest 2022 (Meade, Akerman, et al., 2024). Furthermore, 45% relative humidity was based on outdoor conditions in recent heat waves in Ontario and indoor humidity standards by the American Society of Heating and Air‐Conditioning Engineers (ASHRAE, 2016), with minimal airflow (≤0.3 m/s). The three experimental conditions differed only in the air speed delivered by an electric pedestal fan, which was positioned 1.0 m away, delivering airflow to the front of the body. Participants could eat a self‐provided lunch (∼300 g, with low water content, e.g., peanut butter sandwich) between hours 1.5 and 3.5 of exposure, and which was kept consistent between the three experimental trials. The mass of consumed water and food was measured every hour using a digital kitchen scale. At the end of the exposure, final measurements were recorded and an end‐exposure blood sample collected. Participants then left the chamber and recorded a final nude body mass to enable fluid losses through sweat and urine to be quantified as the change in nude body mass. Net fluid loss was presented as a percentage change from baseline values, corrected for consumed food measured to 1 g using a digital kitchen scale (MK200; Mafiti). Fluid loss was not corrected for fluid consumption or urination, thereby providing an index of total dehydration.

### 2.6 Venous blood collection and sample analysis

Venous blood samples (10 mL) were collected before and at the end of each heat exposure. Participants were seated for ~45 min before sample collection. Samples were transferred directly into plasma (5.4 mg K_2_EDTA) BD Vacutainer tubes and mixed via gentle inversion, and serum separator tubes, respectively. Blood collected in serum separator tubes was allowed to coagulate for ~15 min prior to centrifugation. An aliquot of plasma was used to quantify hematocrit and hemoglobin in duplicate (Ac·T diff2; Beckman Coulter). The remaining blood was centrifuged (1380 × relative centrifugal force) for 10 min before plasma and serum were aliquoted and subsequently stored at −80°C until analysis.

We measured a panel of circulating proteins chosen to evaluate physiological responses to prolonged heat stress and the potential modulatory effects of air flow. Intestinal fatty acid‐binding protein (IFABP), a marker of enterocyte damage, was used to assess potential heat‐induced gastrointestinal epithelial injury. Soluble CD14 (sCD14) and lipopolysaccharide‐binding protein (LBP) are acute phase proteins that indirectly capture innate immune activation and microbial translocation responses – and are often elevated following increases in gastrointestinal permeability. Interleukin‐6 (IL‐6) and tumor necrosis factor‐alpha (TNF‐α) are pro‐inflammatory cytokines commonly elevated during thermal stress and were measured to evaluate systemic inflammation. C‐reactive protein (CRP), a downstream marker of the acute‐phase response, was included to assess broader systemic inflammatory activation. Neutrophil gelatinase‐associated lipocalin (NGAL) was included as an indirect marker of acute kidney stress. Blood samples were collected immediately post‐exposure; however, we acknowledge that some proteins, particularly sCD14 and LBP, may exhibit delayed kinetics and could potentially peak after the sampling time point.

All serum and plasma samples were assessed in duplicate using an enzyme‐linked immunosorbent assay (ELISA). All samples were diluted 1:5 in phosphate buffered saline and 1% bovine serum albumin prior to analysis, apart from CRP and LBP, which were diluted 1:500. All assays (IFABP – DY3078; sCD14 – DY383; LBP – DY870; IL‐6 – DY206; TNFα – DY210; CRP – DY1707; and neutrophil gelatinase‐associated lipocalin, NGAL – DY1757) were performed using a standard sandwich ELISA protocol according to manufacturer specifications and read on a plate reader (SynergyMX, BioTek) at a wavelength of 450 nm with a wavelength correction of 570 nm to minimize the influence of non‐specific wavelength emissions. The inter‐and intra‐plate coefficients of variation were as follows: IFABP 2.55 and 5.64%; sCD14 4.10 and 5.27%; LBP 2.38 and 5.14%; NGAL 5.47 and 3.73%; IL‐6 4.70 and 4.17%; TNF‐α 1.94 and 4.55%; CRP 5.55 and 5.67%. All serum protein concentrations were corrected for changes in plasma volume (Dill & Costill, 1974).

### 2.7 Statistical analysis

All data analysis was performed in R version 4.2.0 (R Foundation). Details on the sample size calculation for the primary outcome, body core temperature, are presented with our original report (O'Connor et al., 2024). As no formal power calculations were conducted for secondary outcomes (i.e., IFABP, sCD14, LBP, TNFα, IL‐6, CRP, and NGAL), these findings should be considered exploratory. The change from baseline for all serum proteins was analyzed via mixed linear models. Condition was modeled as a fixed effect (three levels: control, fan at 2 m/s, and fan at 4 m/s) with baseline data incorporated as a continuous covariate (Vickers, 2001). A random intercept was included for each participant. Contrasts between the control and 2 m/s condition and control and 4 m/s condition are reported, and between condition differences described as the mean difference [95% confidence interval, lower, upper]. A two‐sided *p* ≤ 0.050 was considered statistically significant. No corrections for multiplicity were performed.

## 3 RESULTS

### 3.1 Physiological outcomes

**FIGURE 2:**
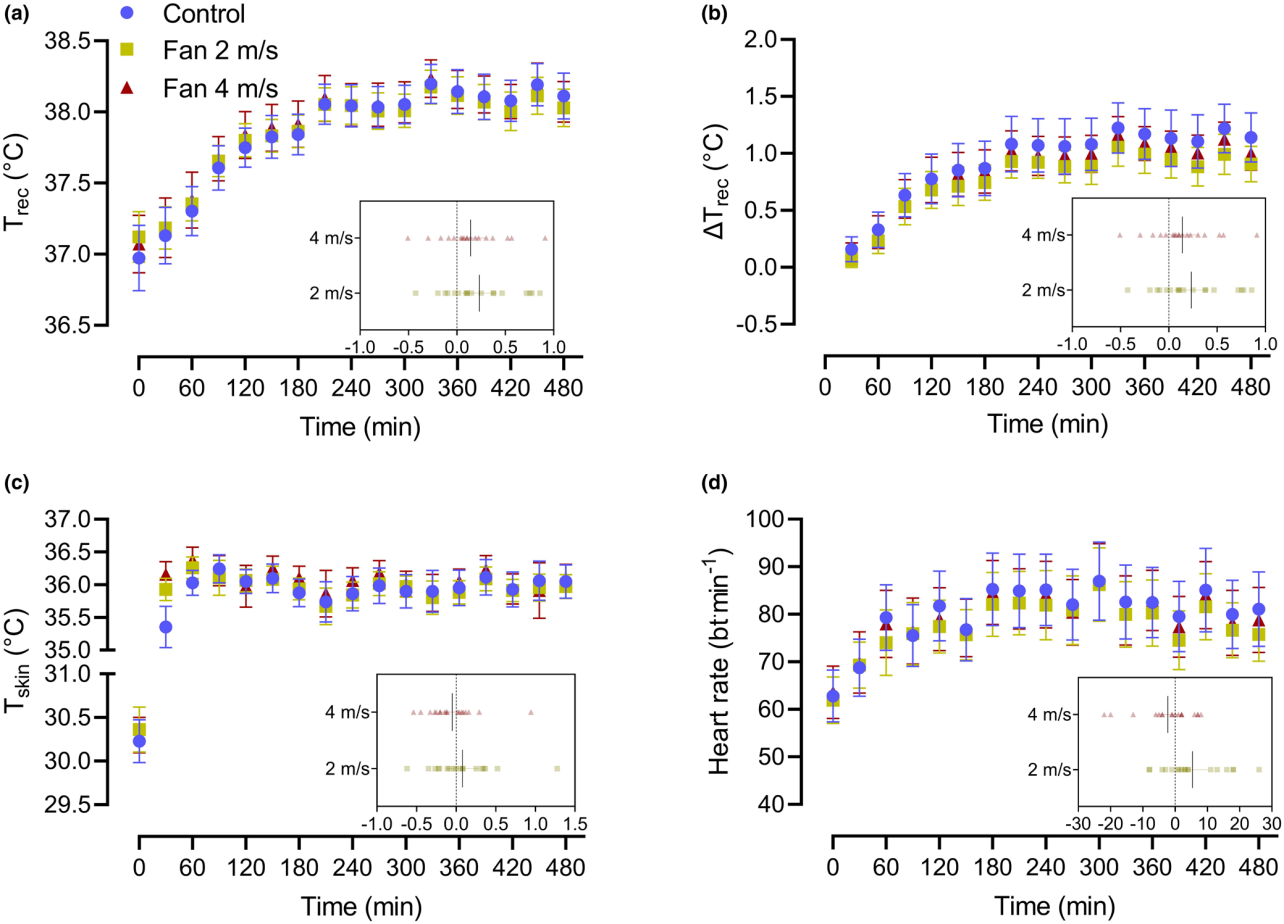
Body core temperature (a), the change in body core temperature (b), mean skin temperature (c) and heart rate (d) throughout the 8‐h heat exposure during the control condition (open white circles), 2 m/s fan condition (light gray squares), and 4 m/s fan condition (dark gray triangles) in 18 older adults. Data show the mean and 95% confidence intervals. The insets illustrate the individual difference in responses between the control and 2 m/s condition, and control and 4 m/s condition. The dashed line demarks the point of no difference, and the cross represents the group mean and 95% confidence interval.

Primary physiological outcome variables for this experiment were previously presented within our original report (O'Connor et al., 2024). Pertinent physiological responses are reported here to provide context for the reader. Body core temperature, the change in body core temperature, mean skin temperature, and heart rate responses throughout each 8‐h heat exposure are presented in Figure 2a–d. End exposure body core temperature increased by 1.1°C (SD 0.4) in the control condition, 0.9°C (SD 0.3) in the 2 m/s condition, and 1.0°C (0.3) in the 4 m/s condition. Peak body core temperatures were 38.3°C (SD 0.3) in the control condition, 38.0°C (SD 0.2) in the 2 m/s condition, and 38.3°C (SD 0.3) in the 4 m/s condition (all *p* ≥ 0.50). Peak heart rate was not different between the control (101 bpm, SD 16) and 2 m/s condition (100 bpm, SD 15; *p* ≥ 0.99), or between the control and 4 m/s condition (102 bpm, SD 16; *p* ≥ 0.99).

Fluid consumption was not different between the control (178, SD 95 mL/h), fan at 2 m/s (183, SD 98 mL/h), and fan at 4 m/s (169, SD 76 mL/h) conditions (all *p* ≥ 0.99). Fluid loss (percent body mass loss) was also not different between the control (−0.2 SD 0.8%) fan at 2 m/s (−0.3, SD 0.8%) and fan at 4 m/s (−0.6, SD 0.6%) conditions (all *p* ≥ 0.14). Consequently, the change in plasma volume was not different between conditions (control 0.7%, SD 4.4); fan at 2 m/s 0.1%, SD 5.5; fan at 4 m/s (0.3%, SD 4.7; all *p* ≥ 0.99).

### 3.2 Biochemical responses

#### 3.2.1 Intestinal epithelial injury and acute phase proteins

**FIGURE 3:**
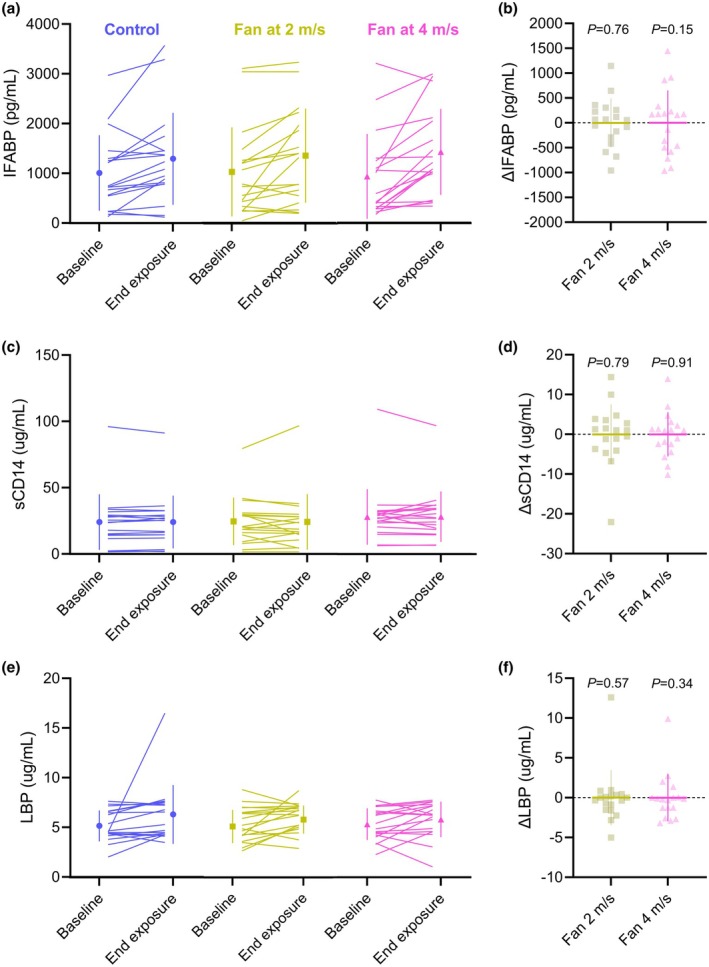
Absolute concentrations of serum intestinal fatty acid binding protein (IFABP; a), soluble cluster of differentiation 14 (sCD14; c) and lipopolysaccharide binding protein (LBP; e) at baseline and at the end of the 8‐h exposure to 36°C, 45% RH. Baseline‐corrected changes in IFABP (b), sCD14 (d) and LBP (f) before and after the control condition, fan at 2 m/s condition, and fan at 4 m/s condition. Solid lines denote individual participant responses. Summary statistics are presented as the group mean and standard deviation. Individual data are shown for 18 participants. The baseline‐corrected end‐exposure changes in IFABP (b), sCD14 (d) and LBP (f) are presented relative to the no fan control condition. Summary statistics are presented as the group mean and standard deviation.

IFABP increased by 364 pg/mL [59, 670] from baseline, regardless of condition (*p* = 0.02). Fan use did not reduce IFABP concentrations relative to the control condition, regardless of air velocity (Figure 3a). The difference between the control and 2 m/s condition was 41 pg/mL [−237, 320; *p* = 0.76], and the difference between the control and 4 m/s condition was 199 pg/mL (−79, 479; *p* = 0.15; Figure 3b).

sCD14 did not increase following the 8‐h heat exposure in any condition (mean 0.97 ng/mL, SD 10.88; *p* = 0.53). Fan cooling did not change sCD14 concentrations relative to the control condition, regardless of air velocity (Figure 3c). The difference between the control and 2 m/s condition was −0.48 ng/mL [−4.0, 3.1; *p* = 0.79], and the difference between the control and 4 m/s condition was 0.19 ng/mL (−3.3, 3.7; *p* = 0.91; Figure 3d).

LBP increased by 3.06 ng/mL [1.12, 5.00] from baseline across conditions (*p* = 0.002). Fan cooling did not reduce LBP concentrations relative to the control condition, regardless of air velocity (Figure 3e). The difference between the control and 2 m/s condition was −0.37 ng/mL [−1.64, 0.90; *p* = 0.57], and the difference between the control and 4 m/s condition was −0.62 ng/mL (−1.89, 0.64; *p* = 0.34; Figure 3f).

#### 3.2.2 Systemic inflammation and renal ischemia

**FIGURE 4:**
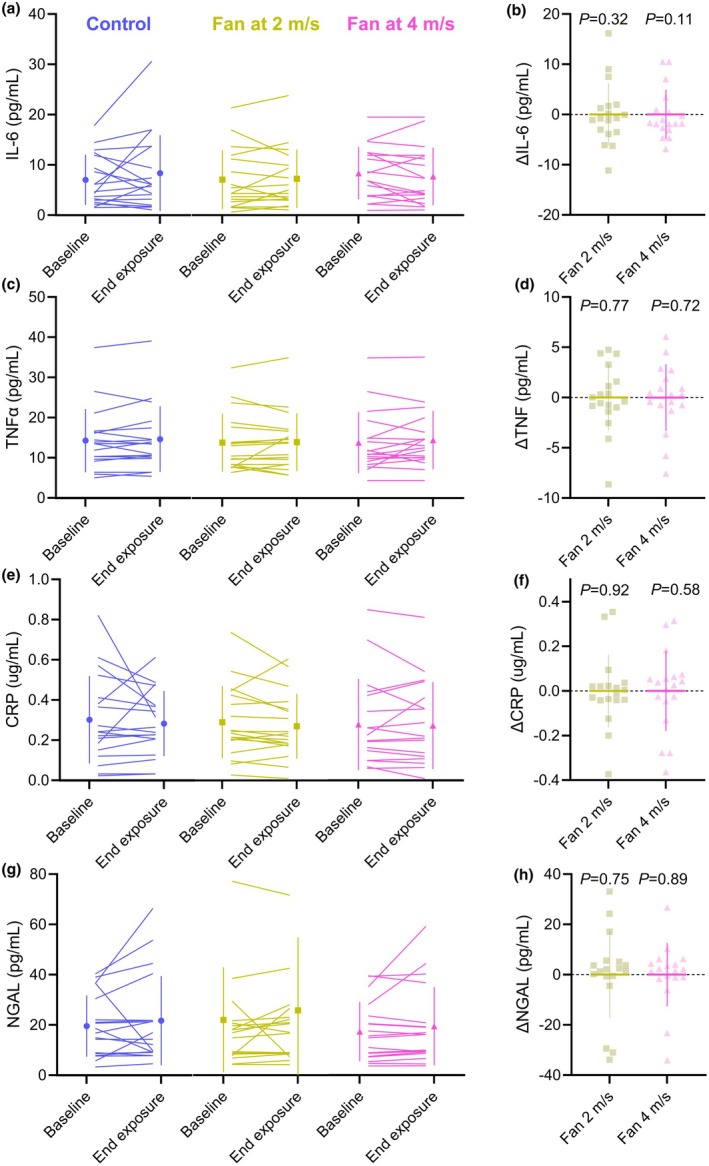
Absolute concentrations of plasma interleukin‐6 (IL‐6; a), tumor necrosis factor alpha (TNFα; c), C‐reactive protein (CRP; e), and neutrophil gelatinase‐associated lipocalin (NGAL; g) at baseline and at the end of the 8‐h exposure to 36°C, 45% RH. Solid lines denote individual participant responses. Summary statistics are presented as the group mean and standard deviation. Baseline corrected changes in IL‐6 (b), TNFα (d), CRP (f), and NGAL (h) before and after the control condition, fan at 2 m/s condition, and fan at 4 m/s condition. Individual data are shown for 18 participants. The baseline corrected end‐exposure changes in IL‐6 (b), TNFα (d), CRP (f), and NGAL (h) are presented relative to the no fan control condition. Summary statistics are presented as the group mean and standard deviation.

IL‐6 did not increase following the 8‐h heat exposure in any condition (mean 1.57 pg/mL, SD 6.86; *p* = 0.15). Fan cooling did not reduce IL‐6 concentrations relative to the control condition, regardless of air velocity (Figure 4a). The difference between the control and 2 m/s condition was −1.18 pg/mL [−0.24, 0.18; *p* = 0.32], and the difference between the control and 4 m/s condition was −1.93 pg/mL (−4.42, 0.57; *p* = 0.11; Figure 4b).

TNFα did not increase following the 8‐h heat exposure in any condition (mean 1.06 pg/mL, SD 4.79; *p* = 0.23). Fan cooling did not reduce TNFα concentrations relative to the control condition, regardless of air velocity (Figure 4c). The difference between the control and 2 m/s condition was −0.22 pg/mL [−1.76, 1.31; *p* = 0.77], and the difference between the control and 4 m/s condition was 0.27 pg/mL (−1.24, 1.78; *p* = 0.72; Figure 4d).

CRP did not increase following the 8‐h heat exposure in any condition (mean 0.04 ug/mL, SD 0.23; *p* = 0.259; Figure 4e). Fan cooling did not reduce CRP concentrations relative to the control condition, regardless of air velocity (Figure 4f).

NGAL, a surrogate marker of renal ischemia, did not increase following 8‐h heat exposure in any condition (mean −1.98 pg/mL, SD 22.65; *p* = 0.54). Fan cooling did not reduce NGAL concentrations relative to the control condition, regardless of air velocity (Figure 4g). The difference between the control and 2 m/s condition was 1.10 pg/mL [−5.87, 8.07; *p* = 0.75], and the difference between the control and 4 m/s condition was 0.49 pg/mL [−6.49, 7.47], (*p* = 0.89; Figure 4h).

## 4 DISCUSSION

We evaluated the impact of electric fan use at two different air velocities on serum proteins associated with intestinal enterocyte damage, systemic inflammation, and a surrogate marker of renal ischemia in older adults following 8‐h heat exposure (36°C, 45% RH). Consistent with other recent studies employing prolonged, acute exposures to extreme heat, we observed a ~364 pg/mL increase in serum IFABP at the end of heat exposure (Foster et al., 2023; Lee et al., 2025a; Lei et al., 2025). We also observed a small ~3.1 ng/mL increase in LBP, an acute phase protein primarily produced by the liver in response to bacterial lipopolysaccharides (Maliszewski & Wright, 1979; Schumann, 1992). Despite the increase in both IFABP and LBP, we observed no increases in sCD14, nor any of the measured inflammatory markers (IL‐6, TNFα, or CRP), nor did we observe any increase in neutrophil gelatinase‐associated lipocalin (NGAL), a surrogate marker of renal ischemia. Importantly, fan use, regardless of air velocity, had no impact on mitigating the increase in both IFABP and LBP during the 8‐h heat exposure in healthy older adults.

The efficacy of electric fans to mitigate dangerous elevations in body core temperature during extreme heat exposure has received considerable attention (Chaseling et al., 2024; Gagnon et al., 2016, 2017; Meade, Notley, et al., 2024; Morris et al., 2021; O'Connor et al., 2024; Ravanelli et al., 2015). Although this cooling modality represents a simple, low‐cost approach for those without access to air conditioning, until recently (O'Connor et al., 2024) their efficacy for heat‐vulnerable groups remains unproven, with limited work addressing the biochemical responses to heat stress with and without fan use. In human models, hyperthermia damages the intestinal barrier and leads to microbial translocation, initiating a cascade of signaling events that can lead to inflammation, and eventually blood coagulation, adverse cardiovascular events, and/or multiple organ failure (Bouchama et al., 2022; Schlader et al., 2022). If electric fan use were shown to be efficacious in mitigating both thermal and cardiovascular strain, it would follow that lesser damage would be experienced at the gastrointestinal barrier.

Our data indicate that fan use did not meaningfully reduce either thermal or cardiovascular strain (O'Connor et al., 2024). Thus, it is not surprising that we observed similar end‐exposure increases in IFABP and LBP across all conditions. Both LBP and sCD14 are acute phase proteins involved in the trafficking of LPS to immune cells; thus, increases in these proteins have been suggested as surrogates for LPS translocation (McKenna et al., 2024). LBP forms an LBP‐LPS complex, facilitating LPS recognition by immune cells via the CD14/toll like receptor 4 (TLR‐4) pathway on immune competent cells (Fang et al., 2002; Kitchens & Thompson, 2003), triggering pro‐inflammatory cytokine release (Chen et al., 2005). Although we observed increases in LBP, we did not see changes in sCD14, findings which are concordant with previous studies using both exercise models (Lee, Flood, et al., 2024) and passive heating exposures (Lee, Russell, et al., 2024; McKenna et al., 2024). The underlying reasons for the increase in one, but not both, proteins are unclear but could be attributable to the different sources of production/release. LBP is produced/secreted by both hepatocytes in the liver and intestinal epithelial cells (Schumann, 1992), whereas sCD14 is produced primarily by immune cells (Maliszewski & Wright, 1979; Wright et al., 1990). In the context of the present study, it could be that heat stress was sufficient to increase LBP production and release locally in the gut via intestinal epithelial cells, or from the liver, but not sufficient to induce immune activation and an increase in sCD14. Regardless of these observations, the change in both IFABP and LBP is far below thresholds thought to be clinically meaningful in the sense of downstream inflammation and systemic inflammatory response syndrome. For example, an increase of >1000 pg/mL for IFABP has been suggested to be clinically relevant regarding downstream microbial translocation and subsequent inflammatory response following exertional heat stress. Similarly, peak LBP concentrations in the range of 200 ug/mL have been observed following thermal injury and/or infectious complications. Whether greater elevations in these systemic proteins would begin to accrue over repeated prolonged exposures (i.e., as a heatwave progresses), thereby leading to downstream inflammatory complications remains to be determined.

We are aware of only one previous study examining the efficacy of fan use in the context of intestinal epithelial injury and downstream inflammation (Lei et al., 2025). In this experiment, fan use with either limited or full fluid replacement was shown to reduce body core temperature but not cardiovascular strain, nor the systemic inflammatory response in otherwise healthy young men exposed to 40°C, 55% RH for 8 h (Lei et al., 2025). During the two fan conditions, participants self‐selected air velocities between ~2–2.5 m/s, which are comparable to our 2 m/s condition. Despite the more extreme heat stress conditions employed by Lei et al., peak body core temperatures during the control condition (i.e., no fan) in younger adults were comparable to those observed across all conditions in the present investigation in older adults (Lei et al., 38.3, SD 0.4°C vs. 38.3, SD 0.3°C herein). Although we observed a greater increase in serum IFABP following heat exposure in our older cohort (~360 pg/mL) relative to the Lei et al. study (~80 pg/mL), we did not observe any downstream systemic inflammatory responses. In contrast, Lei et al. observed marked reductions in body core temperature when electric fans were employed with some fluid replacement and full fluid replacement, with evidence of a systemic inflammatory response seen in all conditions (Lei et al., 2025). Why these authors observed inflammatory responses in otherwise healthy younger adults while we did not observe such responses in healthy older adults is not clear, especially considering the similar end‐exposure body core temperatures and heart rates. Regardless of this discrepancy, our data clearly indicate no benefit of electric fans when the aim is to reduce body core temperature, mitigate cardiovascular strain, and reduce inflammation and end organ damage, although fans do not appear to worsen heat strain either.

Epidemiological data suggest that many heat‐related hospitalizations and deaths in older adults are attributable to renal complications (Bobb et al., 2014; Hopp et al., 2018; Wang et al., 2016). Older adults have been suggested to be at a greater risk for heat‐related renal complications, including an increased risk of acute kidney injury, owing to age‐related alterations in kidney structure and function (Bolignano et al., 2014; Denic et al., 2017). Indeed, older adults have been shown to have augmented increases in plasma creatinine and cystatin C following exposure to very hot dry heat relative to younger adults (McKenna et al., 2024). More recently, the same group observed no differences between younger and older adults in a much broader biochemistry panel designed to assess both kidney function and acute kidney injury (McKenna et al., 2024). In the present investigation, we measured a surrogate marker of renal ischemia (plasma NGAL). We did not see a consistent increase in NGAL following heat exposure, with substantial individual variation across participants (range −27, +52 pg/mL). However, it should be noted that plasma NGAL indicates renal ischemia or glomerular dysfunction but lacks specificity due to potential non‐renal sources (e.g., liver). Future studies should incorporate a broader panel of systemic and urinary biomarkers, thereby identifying the anatomical origin and potential causes of acute kidney injury (AKI)‐related changes (Chapman et al., 2021). For example, urinary IGFBP7 and TIMP‐2, which induce G1 cell cycle arrest in renal epithelial cells during AKI, provide insight into glomerular or tubular damage, with IGFBP7 linked to proximal tubules and TIMP‐2 to distal tubules (Emlet et al., 2017). Both are FDA‐approved for AKI risk assessment (Johnson & Zager, 2018). A comprehensive biomarker panel can thus offer indirect but valuable insight into AKI mechanisms during heat exposure in older adults.

### 4.1 Limitations

There are three limitations that warrant discussion. Firstly, we only assessed the fan interventions under one temperature and humidity combination – designed to be reflective of indoor temperature extremes observed in recent extreme heat events in North America 2022 (Meade, Akerman, et al., 2024). The effects of more extreme temperature/humidity combinations and profiles therefore warrant further study in heat vulnerable groups. Secondly, to maximize ecological relevance, the study was conducted under conditions that would not disrupt the participants normal routines. Participants were instructed to consume their normal breakfast prior to arrival at the laboratory (confirmed via self‐report on arrival) and consumed similarly sized meals for their lunch during each experimental exposure. We accept that differences in macronutrient intake can impact gastrointestinal barrier measurements – however, this has only been demonstrated for prolonged moderate to intensive exercise trials in healthy young adults. It is highly unlikely that large systematic differences occurred between the three experimental conditions since participants were blinded to their condition prior to arrival. Moreover, all our outcome measures were corrected for baseline concentrations during analysis. Finally, this was a secondary analysis of a larger experiment; we were unable to directly measure gastrointestinal permeability; thus, it cannot be inferred that our results are indicative of elevated permeability per se.

### 4.2 Perspectives and significance

As shown in our original report (O'Connor et al., 2024), electric fan use, regardless of air velocity, did not mitigate rises in body core temperature in older adults exposed to simulated indoor overheating. Here we extend upon those findings in showing that fans failed to mitigate increases in serum IFABP and plasma LBP, which occurred to a similar magnitude in all experimental conditions. Despite the increases in both IFABP and LBP, we did not observe subsequent downstream increases in systemic inflammatory markers during a single acute exposure. Although we observed no evidence of a systemic inflammatory response, it is important to note that we only exposed older adults to an acute daylong (i.e., 8‐h) period of heating. Heatwaves last several days, and the impacts of multi‐day exposures on these biochemical responses are not well defined. Whether more prolonged and repeated exposures to heat stress elicit a progressive increase in GI barrier impairment, eventually leading to clinically significant increases in inflammation requires further study.

Considering the entirety of our findings, this project does not provide strong support for the use of electric fans as a standalone cooling modality for heat‐vulnerable older adults under the conditions studied. While future studies are warranted due to only examining a single environmental condition (36°C, 45% RH), it is important to note that these conditions were selected for their ecological relevance, mirroring recent deadly heat wave events in North America (e.g., the 2021 Heat Dome). Our work suggests that other, more powerful cooling interventions (e.g., heat pumps, evaporative coolers) should be prioritized in the protection of heat‐vulnerable older adults during hot weather and extreme heat events. If these options are not available, the use of air‐conditioned spaces (e.g., cooling centres) has been shown to provide robust, transient reductions in body core temperature (Meade, Notley, Akerman, McCormick, et al., 2023), with some evidence of cellular benefits (Lee et al., 2025b; McCormick et al., 2023).

Taken together, electric fan use, whether at or above ecologically valid air velocities (~2 m/s), had no impact on reducing serum IFABP or plasma LBP during an 8‐h prolonged exposure to conditions reflective of indoor overheating observed during hot weather and heatwaves experienced in North America. Given that no clinically relevant effects were observed on thermal and cardiovascular strain, the efficacy of electric fans as a standalone strategy for protecting older heat‐vulnerable adults remains in question.

## FUNDING INFORMATION

This research was funded by the Canadian Institutes of Health Research (grant PJT–1802424) and Health Canada (contract 4500387992) (all funds held by Glen P. Kenny). The funders had no role in trial design, collection, analysis, or interpretation of data, or in manuscript development. No authors received direct compensation related to the development of this article.

## ETHICS STATEMENT

Ethical approval was granted by the University of Ottawa Research Ethics Committee (H‐11‐21‐7572).
